# Clinical efficacy of iodine complex in SARS-CoV-2-infected patients with mild to moderate symptoms: study protocol for a randomized controlled trial

**DOI:** 10.1186/s13063-021-05848-8

**Published:** 2022-01-19

**Authors:** Sohaib Ashraf, Shoaib Ashraf, Moneeb Ashraf, Iqra Farooq, Rutaba Akmal, Muhammad Ahmad Imran, Larab Kalsoom, Sidra Ashraf, Sundas Rafique, Muhammad Ghufran, Muhammad Kiwan Akram, Sohaib-ur-Rehman, Muhammad Faisal Nadeem, Nazish Matti, Uzma Nasim Siddiqui, Ayesha Humayun, Qazi Abdul Saboor, Ali Ahmad, Muhammad Ashraf, Mateen Izhar, DOCTORS LOUNGE consortium, Zaigham Habib, Kanwal Hayat, Ghazala Amjad, Misbah Kousar, Shahroze Arshad, Umair Hafeez, Tayyab Mughal, Muhammad Sikandar Saleem, Ammara Ahmad, Abeer bin Awais, Noman Khalid, Qurat-ul-Ain Iqbal, Muhammad Hassan, Abdul Rehman Virk, Mehak Gul, Muhammad Tayyab Naeem, Roa Umer, Musa Khalil, Tayyaba Muzafar, Sibgha Zulfiqar, Saadia Shahzad Alam, Emed Chohan, Muhammad Imran Anwar, Ali Rafique Mirza, Amber Malik, Talha Mahmud, Adeen Akmal, Syed Sami Husain Sherazi, Zartasha Safdar, Sohail Ahmad, Ali Arshad, Khawar Nawaz, Muhammad Ismail Khalid Yousaf, Muhammad Nauman Zahid

**Affiliations:** 1 Department of Cardiology, Shaikh Zayed Post-Graduate Medical Institute, Lahore, Pakistan; 2 Department of Pathophysiology, Riphah International, Lahore, Pakistan; 3 Department of Pharmacology, Mayo Hospital, Kingedward Medical University, Lahore, Pakistan; 4 Department of Paediatric Surgery, Children Hospital, Lahore, Pakistan; 5 Department of Medicine, Sahara Medical College, Narowal, Pakistan; 6 Department of Microbiology, Shaikh Zayed Post-Graduate Medical Institute, Lahore, Pakistan; 7 Department of Medicine, Services Institute of Medical Sciences, Lahore, Pakistan; 8 Department of Biochemistry, University of Veterinary and Animal Sciences, Lahore, Pakistan; 9 Department of Oncology, Mayo Hospital, Kingedward Medical University, Lahore, Pakistan; 10 ESACHS (Empresa de Servicio Externo de la Asociación Chilena de Seguridad), Santiago, Chile; 11 Department of Animal Nutrition, University of Veterinary and Animal Sciences, Lahore, Pakistan; 12 Department of Community Medicine, Shaikh Khalifa Bin Zayed Al-Nahyan Medical and Dental College, Lahore, Pakistan; 13 The University of Strasbourg, Alsace, France; 14 University of Veterinary and Animal Sciences, Lahore, Pakistan; 15 Department of Pharmacy, Quaid-i-Azam University, Islamabad, Pakistan; 16 Department of Medicine, Shaikh Zayed Post-Graduate Medical Institute, Lahore, Pakistan; 17 Department of Microbiology, Infectiology and Immunology, Centre Hospitalier Universitaire (CHU) Sainte Justin/University of Montreal, Montreal, Canada; 18 Department of Pharmacology and Toxicology, University of Veterinary and Animal Sciences, Lahore, Pakistan

**Keywords:** Iodine, COVID-19, SARS-CoV-2

## Abstract

**Background:**

Coronavirus disease 2019 (COVID-19) caused by the novel coronavirus-infected millions globally. Despite a wide range of advised options for the treatment of COVID-19, a single strategy to tackle this pandemic remains elusive, thus far. That is why we are conducting a clinical trial to find out the efficacy of iodine complex to clear a viral load of severe respiratory syndrome coronavirus-2 (SARS-CoV-2) along with a reduction in time taken to alleviate symptoms.

**Method:**

The proposed study is a placebo-controlled, add-on, randomized trial using parallel group designs. This is a closed-label and adaptive with sample size reassessment, multi-centered design with a 1:1:1:1 allocation ratio and superiority framework. It will be conducted in Shaikh Zayed Post-Graduate Medical Complex, Ali Clinic, and Doctors Lounge, Lahore, Pakistan. This study will have three arms of mild to moderately symptomatic COVID-19 patients (50 patients in each) which will receive ionic-iodine polymer complex with 200 mg of elemental iodine: interventional arm A will have encapsulated, arm B will receive suspension syrup form, arm C will get throat spray, while arm X will be standard care with placebo. Data will be collected on self-constructed, close-ended questionnaires after obtaining written consent. Data will be analyzed using SAS version 9.4. COVID-19 patients will be monitored by RT-PCR and HRCT (high-resolution computed tomography) chest. In addition to these, the duration of the symptomatic phase and mortality benefits will be analyzed in both groups.

**Discussion:**

The study is designed to measure the superior efficacy of the iodine complex as an add-on in treating COVID-19-positive patients with mild to moderate symptoms. This combination is hypothesized to improve various parameters like rapid viral load reduction, clinical and radiological improvement, lower mortality, and reduction in hospitalization. The trial will aid in devising a better strategy to cope with COVID-19 in a relatively inexpensive and accessible way. The implications are global, and this could prove itself to be the most manageable intervention against COVID-19 especially for patients from limited-resource countries with deprived socioeconomic status.

**Trial registration:**

ClinicalTrials.govNCT04473261. Registered on July 16, 2020.

## Administrative information

**Table:** 

Title {1}	Clinical efficacy of iodine complex in SARS-CoV-2 infected patients: study protocol for a randomized controlled trial
Trial registration {2a and 2b}.	Clinical Trial registration is at www.clinicaltrials.gov with ID **NCT04473261** dated July 16, 2020.
Protocol version {3}	Protocol Version Number is 2.4 dated 10/02/2021
Funding {4}	Shaikh Zayed Post-Graduate Medical Complex and Smile Welfare Organization
Author details {5a}	oSohaib Ashraf Department of Cardiology, Shaikh Zayed Post-Graduate Medical Institute, Lahore, Pakistan. sohaib@skzmdc.edu.pk oShoaib Ashraf Department of Pathophysiology, Riphah International, Lahore, Pakistan. shoaib.ashraf@mail.mcgill.ca oMoneeb Ashraf Department of Pharmacology, Mayo Hospital, Kingedward Medical University, Lahore, Pakistan. moneeb-ashraf@hotmail.com oIqra Farooq Department of Paediatric Surgery, Children Hospital, Lahore, Pakistan. iqrafarooq93@gmail.com oRutaba Akmal, Department of Community Medicine, Sahara Medical College, Narowal, Pakistan. rutabaakmal@gmail.com oMuhammad Ahmad Imran Department of Microbiology, Shaikh Zayed Post-Graduate Medical Institute, Lahore, Pakistan. ahmad.imran@skzmdc.edu.pk oLarab Kalsoom Department of Medicine, Services Institute of Medical Sciences, Lahore, Pakistan. laraibshah4853@gmail.com oSidra Ashraf, Department of biochemistry, University of veterinary and animal sciences, Lahore, Pakistan sidra.ashraf@uvas.edu.pk oSundas Rafique, Department of West Medicine, Mayo Hospital, Kingedward Medical University, Lahore, Pakistan. drsundasr8@gmail.com oMuhammad Ghufran, ESACHS (Empresa de Servicio Externo de la Asociación Chilena de Seguridad), Chile, dr.ghufran56@gmail.com oMuhammad Kiwan Akram, Department of animal nutrition, University of veterinary and animal sciences, Lahore, Pakistan. kiwan.akram@uvas.edu.pk oSohaib-ur-Rehman, Department of Community Medicine, Shaikh Khalifa Bin Zayed Al-Nahyan Medical and Dental College, Lahore, Pakistan. sohaib.ur.rehman@outlook.com oMuhammad Faisal Nadeem, The University of Strasbourg, Alsace, France; University of Veterinary and Animal Sciences, Lahore, Pakistan. faisal.nadeem@uvas.edu.pk oNazish Matti, Department of pharmacy, Quaid-i-Azam University, Islamabad, Pakistan. nazish341@yahoo.com oUzma Nasim Siddiqui Department of Medicine, Shaikh Zayed Post-Graduate Medical Institute, Lahore, Pakistan. uzmamamoon@gmail.com oAyesha Humayun Department of Community Medicine, Shaikh Khalifa Bin Zayed Al-Nahyan Medical and Dental College, Lahore, Pakistan. drayeshah@gmail.com oQazi Abdul Saboor Department of Cardiology, Shaikh Zayed Post-Graduate Medical Institute, Lahore, Pakistan. drsaboor04@gmail.com oAli Ahmad# Department of Microbiology, Infectiology and Immunology, Centre Hospitalier Universitaire (CHU) Sainte Justin/University of Montreal, Montreal, Canada. ali.ahmad@recherche-ste-justine.qc.ca oMuhammad Ashraf Department of Pharmacology and Toxicology, University of veterinary and animal sciences, Lahore, Pakistan. drashraf2001@uvas.edu.pk oMateen Izhar Department of Microbiology, Shaikh Zayed Post-Graduate Medical Institute, Lahore, Pakistan. mateen@cantab.net oDOCTORS LOUNGE consortium#, sohaib-ashraf@outlook.com
Name and contact information for the trial sponsor {5b}	Syed Kazam Ali, kazimformanite@gmail.com
Role of sponsor {5c}	There will not be any influential role of sponsor/funder in study design; collection, management, analysis, and interpretation of data; writing of the report; and the decision to submit the report for publication.

## Introduction

### Background and rationale {6a}

As the world was still recovering from The Great War, mayhem which shook the world and killed more than 40 million soldiers and civilians, mother earth suffered from a pandemic of influenza. This deadly virus, in a year, spread everywhere on earth and diseased roughly 500 million and killed 100 million people. Surprisingly after the 101st anniversary of that horrendous plague, we are again witnessing a fast-spreading, highly infectious severe respiratory syndrome coronavirus-2 (SARS-CoV-2) sweeping across the continents. This disease first originated in Wuhan, China, in December 2019 which was later called coronavirus disease 2019 (COVID-19). The illness was declared a pandemic by the World Health Organization (WHO) in March 2020 [1, 2]. Despite being huge data available on the virus epidemiology, pathophysiology, virology, diagnosis, prevention, and management, still most of the available management options are unable to provide promising results and this demands a dire need to find a cure for this highly contagious virus.

Despite this wide range of options available for the treatment of COVID-19, none has been proven to be a definitive therapy against this virus. This makes it the need of the hour to think outside the box and prescribe newer formulations and conduct trials for treating COVID-19. This makes it the need of the hour that a novel idea of using micronutrients should also be proposed as an antiviral in this trial.

Micronutrients are also essential for the body to produce enzymes, hormones, and other substances essential for proper growth and development [3]. Their deficiencies have been reported in many diseased states. Diet deficient in these nutrients may lead to compromised humoral and cell-mediated immunity [4]. Iodine is considered an excellent antimicrobial and specifically antiviral action of the elements commonly used in nutrition [5–7]. Cell culture studies also show that higher concentrations of iodine have exceptional antiviral activities. The potential of iodine against human immunodeficiency virus (HIV) has been tested due to its powerful antiviral activity [8]. It highlighted some important aspects of the mechanism of action of iodine as an antiviral agent [9]. Moreover, through scientific studies, it has been established that iodine complexes circulate throughout the body in the extracellular fluids found between the cells of the body. If cell surface proteins have the amino acid tyrosine on the outside, the passing iodine complex reacts with this tyrosine. This reaction denatures the proteins and thus kills the abnormal cell. Intra-membrane proteins may have tyrosine which is only exposed when the membrane is distorted by abnormal cell development. So, the iodine complex supports the surveillance system for removing abnormal cells from our bodies. Iodine complex inactivates viruses by interfering with the protein coat of the virus hence the ability of the viruses to adsorb to host cell is impaired. Iodine complex also triggers a mechanism for apoptosis (normal programmed death of cells as part of their life cycle) a process for destroying cells that present a threat to the integrity of the organism, like cells infected with viruses [9].

Potassium iodide boosts not only the humoral immune system of the body by increasing immunoglobulin production but also increases peripheral lymphocytes level which is important in the host response against viruses. Supplementation of potassium iodide is essential for enhancing humoral immunity against pathogens [10].

In vitro studies were conducted on the antiviral properties of iodine complex against severe respiratory syndrome coronavirus-2 (SARS-CoV-2) at the University of Veterinary and Animal Sciences, Lahore, Pakistan, which showed strong to moderate antiviral activity [11]. Another in vitro trial has suggested the antiviral activity of povidone-iodine against SARS-CoV-2 when exposed for more than 60 s and can be used in various concentrations in oral and nasal formulations [12]. Consistent with that is an in vitro study published in *JAMA* showed that the use of povidone-iodine at different concentrations successfully deactivated the COVID-19 virus in only 15 s as compared to 70% ethanol used as control which failed to do so [13]. This was also observed in the past in SARS-CoV and Middle Eastern Respiratory Syndrome (MERS) in which iodine-containing preparations showed the same virucidal effect as 70% ethanol as control [14].

With the unsettling trend of coronavirus worldwide, it is considered to evaluate iodine complex as a potentially affordable and accessible remedy to mitigate infection [15].

### Objectives {7}

The objective of the study is to measure the efficacy of the ionic-iodine polymer complex in reducing the length of the symptomatic phase along with earlier SARS CoV-2 clearance and radiologically better chest as compared to the placebo group in mild to moderately symptomatic patients.

### Trial design {8}

This is a placebo-controlled, multi-armed, add-on to standard therapy, interventional, randomized trial using a parallel group design. It is closed-labeled, adaptive with sample size reassessment, and multi-centered with a 1:1:1:1 allocation ratio design with a superiority framework.

## Methods: participants, interventions, and outcomes

### Study setting {9}

Clinical sampling will be done from Shaikh Zayed Post-Graduate Medical Complex, Ali Clinic and Doctors Lounge, Lahore, Pakistan.

### Eligibility criteria {10}

All COVID-19 diagnosed and declared non-pregnant adults with mild to moderate illness will be recruited in the study. Patients with acute or chronic ailments, who are breastfeeding, or with history of iodine allergy will be excluded from the trial. Patients not giving consent will also be eliminated.

### Who will take informed consent? {26a}

The site investigator will take written informed consent from all trial participants by giving them the specifically constructed informed consent form. The consent will be taken on the recruitment site after explaining the procedure to the participants in detail.

### Additional consent provisions for collection and use of participant data and biological specimens {26b}

Site investigators will be responsible for taking consent regarding every other matter if required. An informed consent form will contain a section on permission to draw and conduct specified tests on blood samples and conduct radiological investigations as per the protocol of the study.

## Interventions

### Explanation for the choice of comparators {6b}

All patients will be assigned in 4 arms at the time of randomization. Placebo comparator group, arm X, will receive a placebo empty capsule, in addition to standard care. Interventional arms A, B, and C will be given ionic-iodine polymer complex (200 mg of elemental iodine) in 3 different formulations (capsule, syrup, and nasal spray, respectively). These formulations are being tested for comparative effectiveness and evaluation of the best possible route of administration.

### Intervention description {11a}

In this multi-armed study, ionic-iodine polymer complex will be given using three formulations with 200 mg of elemental iodine to evaluate efficacy for a maximum of 14 days or when the patient is fully recovered.

- Arm A will be receiving 200 mg iodine complex capsule three times a day
- Arm B will be receiving iodine complex suspension syrup form 40 ml three times a day
- Arm C will be receiving iodine complex nasal spray 2 puffs three times a day.
- Arm X will be receiving a placebo of empty capsule three times a day.

All patients will receive standard care as per version 3.0 of clinical management guidelines for COVID-19 established by the Ministry of National Health Services of Pakistan COVID-19 guidelines of the study setting.

### Criteria for discontinuing or modifying allocated interventions {11b}

Fixed doses will be given throughout the study and interventional drug administration will be stopped immediately in following conditions.

1. Patient becomes severely symptomatic during trial conduction.
2. Any adverse drug reaction.
3. Organ failure secondary to any administered drug.
4. Denies/backs off from further participation.

Regardless of any of these conditions, the participant’s data will be retained and analyzed in the trial to follow-up and prevent any loss of data.

### Strategies to improve adherence to interventions {11c}

To improve adherence to the intervention, participants will be counselled about the advantages of this study. All the participants will be monitored regarding compliance to their assigned treatment strategy and health professionals will administer the drugs. As this trial will include quarantined patients, hence direct observational method will be sufficient to make sure compliance and adherence to the interventions.

### Relevant concomitant care permitted or prohibited during the trial {11d}

As per hospital protocol (study setting), the care and interventions permitted will be used and no specific prohibited care is in this trial. As far as drug reactions are concerned, they will be treated by health care workers on the spot and will be reported afterwards.

### Provisions for post-trial care {30}

No post-trial care will be needed in our study setup as half-lives of the administered drugs are within hours to days.

### Outcomes {12}

Primary outcomes will include time to SARS-CoV-2 RT-PCR clearance, alleviation of symptoms, and better high-resolution computerized tomography (HRCT) chest score. Thirty-day mortality will be used as a secondary outcome. SARS-CoV-2 RT-PCR will be done on admission day (0 day) and then after every 4th day for 12 days or till the symptoms are resolved and RT-PCR gets negative. Symptom resolution is defined by the resumption of mild to moderate symptoms COVID-19 symptoms (defined by the definition of symptoms). RT-PCR will only be shown as positive or negative (as a limitation of our study of not getting the viral load). Time taken for the alleviation of symptoms will be measured as the number of days the patient remained symptomatic, i.e., the difference between onset of COVID-19 symptoms to complete resolution. HRCT chest scoring will be done as follows: Lungs divided into five lobes and each lobe is scored as 0 (no involvement), 1 (< 5% involvement), 2 (up to 25% involvement), 3 (26–49% involvement), 4 (50–75% involvement), and 5 (> 75% involvement). The overall HRCT chest score will be from 0 (no involvement) to 25 (maximum involvement) [16]. The HRCT findings are described via standard international terms, which are classified by the Fleischner Society glossary with peer-reviewed literature on viral pneumonia. The terms being used are ground glass opacity (GGO), crazy-paving pattern, and consolidation [17, 18]. Like a previous study [19], a maximum of 4 HRCT chest will be performed starting from day 0 followed by every fourth day till patients’ PCR become negative.

### Participant timeline {13}

**Fig. 1:**
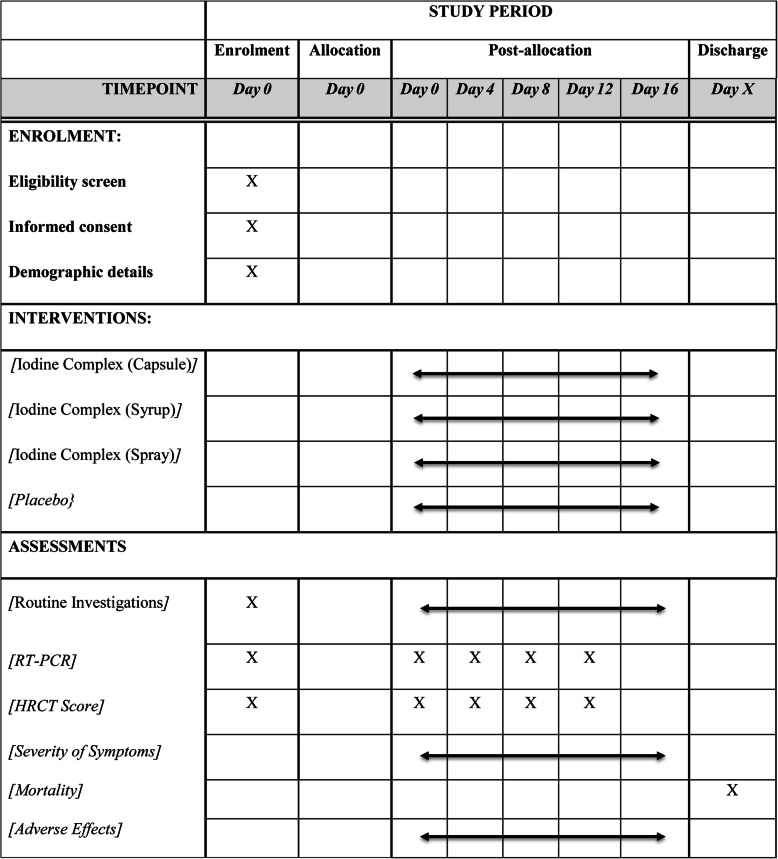
Participant timeline during study period

Schedule of enrolment, interventions (including any run-ins and washouts), assessments, and visits for participants have been shown in a schematic diagram (Fig. 1).

### Sample size {14}

**Fig. 2:**
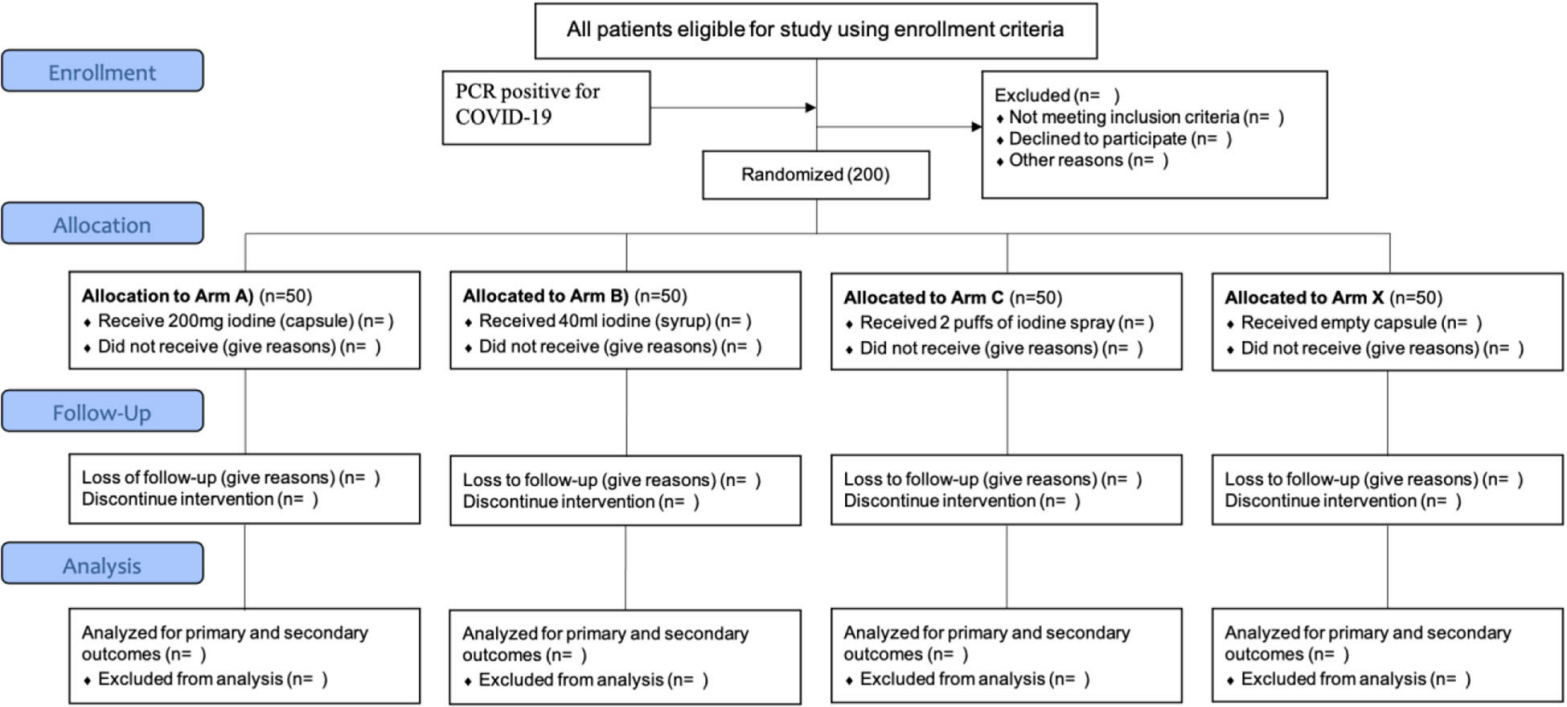
CONSORT flow diagram

Considering it a novel idea to use iodine in the management of COVID-19, no software was used to calculate the sample size (Fig. 2). However, arbitrarily 50 patients in each arm with a total of 200 patients sample size for a multi-centered study in Pakistan will be randomized. In this adaptive trial, sample size reestimation is planned to achieve the study objectives, if needed. Time taken for the elevation of the symptoms will be considered as relevant endpoint (RE) while SARS-CoV-2 RT-PCR clearance and HRCT chest score will be studied as additional endpoints (AE). The required sample size will be calculated with 0.05 alpha and 80% power of the study.

### Recruitment {15}

Recruitment will be done in Shaikh Zayed Medical Complex, Ali Clinic, and Doctors Lounge with mild to moderate symptomatic patients. All patients’ records will be analyzed at the center and eligible participants will be separated. All the eligible participants will be assessed according to our inclusion and exclusion criteria. Fully equipped site investigators with full precautions will do all these proceedings. At the start of the study trial, all recruited participants must provide a written informed consent form as per the plan to get them enrolled for the study.

## Assignment of interventions: allocation

### Sequence generation {16a}

Randomization will be done using lottery method. As patients might be admitted at different times so they will be recruited after taking written informed consent (following all standard protocol for infection control and disinfection) and will be randomized by selecting a slip from the box containing 50 slips of each arm labeled as A, B, C, and X. Arms A, B, and C will be the add-on interventional arms while arm X will be the placebo arm.

### Concealment mechanism {16b}

The allocation sequence will be computer generated that will be concealed from site investigators, allocated participants, and treatment providers until the final interventional allocation is done. However, the sequence allocation will be conveyed telephonically to the respective investigator to allocate the patient in either group. Implementation of the intervention will be done by the help of co-investigators not involved in assessing clinical outcomes.

### Implementation {16c}

The therapists will have no influential role in study outcomes and analysis while only disclosing the treatment plans to patients. Local site investigators and other study members that are involved in participant’s enrolment will not be allowed to receive allocation information to prevent study bias. Meanwhile, generation of allocation sequence and assignment of the intervention will be done by co-investigators.

## Assignment of interventions: blinding

### Who will be blinded {17a}

Trial participants, care providers, outcome assessors, and data analysts are blinded, respectively, by using placebo group, by using site investigators to provide placebo or drugs to participants, by using blinded clinicians to assess the clinical outcome and laboratory or radiological findings, and by using analysts from other institution that are not having any conflict of interest in research. The details will be announced after locking the data in a database sheet at the end of the trial.

The study experimental drug and placebo will be prepared by the team of pharmacists and pharmacologists in the Department of Pharmacology and Toxicology, University of Veterinary and Animal Sciences, Lahore, Pakistan. Both drugs will look alike, as they will have same packing but unique randomization codes. Participants, site investigators, care providers, outcome assessors, study coordinators, data managers, and statisticians will be blinded, and blinding codes will be revealed at the end of this study.

### Procedure for unblinding if needed {17b}

Unblinding is permissible if the patient develops severe symptoms and needed extra treatment by revealing a participant’s allocated intervention during the trial. If unblinding is required, the trial managers and data coordinators will have access to group allocations and any unblinding will be reported.

## Data collection and management

### Plans for assessment and collection of outcomes {18a}

Microsoft Access, a database management system (DBMS) of Microsoft Office, will be used to ensure the data safety. SARS-CoV-2 RT-PCR test and HRCT score will be done every fourth day while COVID-19 symptoms evaluation will be done on daily basis to access the time taken for the alleviation of symptoms. Thirty-day mortality will be assessed by the number of deaths per group after 30 days of randomization in all groups. All trial data will be collected by site investigators and two site investigators will enter the data, recheck twice for possible errors separately, and make certain its integrity. Pre-approved questioner regarding demographics of the patient and data collection will be used while all the testing will be done by International Organization for Standardization (ISO) certified laboratory (Shaukat Khanum Laboratory). Principal investigators will visit twice weekly the study site. Trial steering committee members will make unexpected and unplanned visits as well. There is no conflict of financial and non-financial interest with sponsors and researchers.

### Plans to promote participant retention and complete follow-up {18b}

All the participants will be ensured their safety and will be guided about the study conduction and its beneficial outcomes. This study will be conducted till the patients hospitalized or home-quarantined in homes test negative for SARS-CoV-2 RNA in two consecutive nasopharyngeal swabs done using RT-PCR. All the data will be collected while the patient is already admitted in the hospital except 30-day mortality. Contact numbers and addresses of all participants will be reported at the start of study for which can be used, if needed, after written consent is obtained from the patient. Follow-up will be done using phone numbers of the patients to access the mortality benefit of intervention.

### Data management {19}

Participants IDs will be used for confidentiality purposes and these IDs will be linked to demographic information securely and separately. The final data set of RCT will have coded data and can only be assessed by principal investigators. Data coding will be done by allocating medical record numbers. Data entry will be done electronically once paper-based case report form (CRF) has been received at the trial office. Once the data is entered, CRF will be destroyed to ensure safety and security. Data collection from the site and data entry into the database will be done by a different set of trained site investigators. All outcomes will be double-checked by the researchers prior to data collection and data storage. To ensure data’s integrity and safety, various meetings by the research team will be conducted on a regular basis.

### Confidentiality {27}

In addition, confidentiality of participants’ data is ensured by using participants’ IDs rather than identifiable information in the dataset (i.e., coding) and by storing the document linking the IDs to the identifiable information separately and securely.

### Plans for collection, laboratory evaluation, and storage of biological specimens for genetic or molecular analysis in this trial/future use {33}

Trained staff will collect nasopharyngeal swab samples as per biosafety and personal safety guidelines of World Health Organization. Samples will be maintained at − 80°. Patients’ follow-up will be done daily by the investigators while PCR will be repeated every 4th day for monitoring of primary end point (measuring time to return for COVID19 RT-PCR test to return negative). Patients will be evaluated clinically on daily basis; relevant investigations will be repeated as needed.

## Statistical methods

### Statistical methods for primary and secondary outcomes {20a}

Mean ± S.D will be used for quantitative data and *f*(%) will be used for categorical data. Frequency and percentages will be measured for categorical data. Data normality will be checked using the Shapiro Wilks test. If data is normal, independent sample *t*-test will be used to compare quantitative outcome such as mean hospital stay; otherwise, Mann Whitney *U* test will be used to compare median of these quantitative data. Chi-square *t*-test/ Fisher’s exact test will be applied to compare severity of symptoms and outcome (discharge or mortality), etc. For follow-up analysis, Wilcoxon test will be applied. If data supports the necessary assumptions of time to event data, survival analysis/ Kaplan Meier test will be applied. Method of analysis planned to be used in primary and secondary outcomes are mentioned in Table 1. *P* value ≤ 0.05 will be considered as significant.

**Table 1:** Primary and secondary outcomes

Outcome	Hypothesis	Outcome measure	Method of analysis
**Primary outcomes**			
1. Time taken for alleviation of symptoms	Earlier symptom resolution	Yes/No	Multivariant analysis, cox proportional hazards models
2. Time taken for viral load clearance	Earlier RT-PCR negative	Positive/ negative	
3. HRCT chest score at day 4	Better score resolution	Score from 0 to 25	Mixed Ordinal Logistic Regression Model
**Secondary outcomes**			
Mortality	Lower	Yes/No	Fisher exact test

### Interim analyses {21b}

The risk aptitude for this study is customized as low risk, considering the use of nutraceutical product. However, as it involves vulnerable COVID-19 patients and novel drugs are being tested for repurposing, strict safety measurements will be taken. As a part of our safety measurements, the investigator, Qazi Abdul Saboor (Professor of Cardiology, SZH, Lahore), with the biostatistician, Prof. Dr. Muhammad Azam (Dean faculty of biostatistics, UVAS, Lahore), will conduct an interim analysis. The prime focus of this analysis will be mortality and incidence of any serious adverse effects. This will be carried out after randomization and inclusion of half of the patients. For these specific outcomes, those conducting the interim analysis will be unblinded. No stopping rules for the primary endpoint have been defined as this is the first trial of its kind.

### Methods for additional analyses (e.g. subgroup analyses) {20b}

Adjusted and subgroup analysis may be applied as per the biostatistician, if needed. In that case, both unadjusted and adjusted analyses are provided along with the main analysis.

### Methods in analysis to handle protocol non-adherence and any statistical methods to handle missing data {20c}

The intention-to-treat analysis set will be used to test the superiority. All patients will be considered as randomized despite receiving the randomized treatment as per our anticipation. Reasons for each group’s randomization and withdrawal will be reported and compared qualitatively and sensitivity analysis (augmented data) is being used to overcome the effect of any missing data on results. The participants who withdraw consent for continued follow-up (Dropouts) will be assessed by modern imputation methods for missing data

### Plans to give access to the full protocol, participant-level data, and statistical code {31c}

Only the principal investigators will have access to the full trial dataset to ensure that the overall results are not disclosed by an individual of trial prior to the main publication. Grant public access to the full protocol, participant-level dataset, and statistical code will be given through publication.

## Oversight and monitoring

### Composition of the coordinating center and trial steering committee {5d}

Coordinating center and trial steering committee will be comprised of medical lab technologist (Sidra Ashraf, PhD), clinical pharmacist (Faisal Nadeem, PhD), clinical pharmacologists and toxicologist (Moneeb Ashraf, PhD), virologist (Mateen Izhar, PhD), immunologist (Ali Ahmad), biostatisticians (Muhammad Azam, PhD), epidemiologist (Ayesha Humayun, PhD), and consultants of medicine (Dr. Uzma, MBBS), pulmonology (Talha Mahmud, MD), and cardiologist (Amber Malik, MBBS). This committee will be responsible for the safety, trial safety, and dosage calculation to get results of endpoints. This will have all the authority to stop the clinical trial all together. Data management team will be comprised of principal investigators, co-investigators, and site investigators. This team will be responsible to ensure the execution of the clinical trial in best possible way as defined by the study protocol. Site investigators are responsible for data collection and quality check of data at collection points/ study setting. Kiwan Akram will manage data compilation, cleaning, editing, and entry on SAS along with a biostatistician.

### Composition of the data monitoring committee, its role and reporting structure {21a}

Data monitoring committee (DMC) has a biostatistician (Muhammad Azam, PhD), an epidemiologist (Ayesha Humayun, PhD), a microbiologist (Mateen Izhar, PhD), a pulmonologist (Talha Mehmood, MD), and principal investigators (Muhammad Ashraf, PhD and Sohaib Ashraf, MBBS) in it. There is no financial or non-financial conflict of interest as the committee will be independent of sponsor and competing interests.

### Adverse event reporting and harms {22}

The researchers will record any adverse, unpredictable, or undesirable sign and symptom and it will be discussed with the care providers. A comprehensive evaluation will be conducted to evaluate the co-relation between experimental drug and the developing signs and symptoms. The investigator will respond appropriately to ensure the wellbeing of the patient in case of any unforeseen event and all the details will be written carefully. Moreover, regular follow-up will be made certain until the patient regains his/her health. If the adverse event happens during the study intervention will be reported Institutional Review Board (IRB).

### Frequency and plans for auditing trial conduct {23}

Weekly audit will be done by principal investigators. Monitors will audit by visiting trial sites while performing and resolving solutions to various problems. The monitor will verify the following variables for all patients on every visit: biodata of participants, signed informed consent, eligibility criteria, date of randomization, group allocation, treatment assigned, and adverse events if any. Auditing of the clinical trial will be done by trail steering committee in weekly zoom meeting where all the audits will be provided by the site investigators and research coordinators.

### Plans for communicating important protocol amendments to relevant parties (e.g., trial participants, ethical committees) {25}

To modify protocols including eligibility criteria and outcomes, permission will be needed to get approved by the trial steering committee and the notification will be done to relevant parties including IRB trial registry. All the plans about any amendments in our trial will be communicated to the trial site staff in person.

### Dissemination plans {31a}

The publication subcommittee will review the publication, all the endpoint data, primary outcome analysis, and the study results and recommend the changes to the author. After the changes are done, recommendations are finally submitted to the steering committee for approval. Study results will be disclosed to all study participants, member physicians, patients, and other medical personnel.

## Discussion

Although the vaccines are showing success, but more than a year has passed yet no cure for COVID-19 is available and most of the treatment relies on supportive measure. Here the role of iodine comes, as it solutions have been long used as microbicidal agents and they offer appropriate safety profile [20]. The study shows that patients treated with oral formulations of iodine show better prognosis than the placebo hence establishing its role in treating the disease. It has also been hypothesized that less number of deaths seen in Japan despite boosting a large number of old age population is because of the role of iodine in supporting innate immunity against viral pathogens since Japanese are famous for taking higher amounts of iodine [21] Since different preparations have different concentration-dependent efficacy and side effects, this will provide us to a better idea of optimal dosage with better efficacy and limited toxicity. The efficacy of iodine complex which will be reported in our trial will be compared to those of other topical alternates used to stop viral growth in nasopharynx as published in literature to establish the superiority, if any, of the iodine-based preparations. As previous studies have some concentration-dependent damage by iodine complexes to normal mucociliary mechanism and nasal epithelium [22, 23], hence, this study will look for such possible adverse outcomes keeping regular follow-up. Possible lowering of efficacy of iodine preparation will be looked for as nasal secretions, debris, and poor nasal hygiene dilute the concentration of the formulations and reduce its penetrance.

The study will be limited to viral load clearance rather than measuring viral load reduction. Another limitation would be the relatively smaller number of patients to establish clinical efficacy. Larger randomized clinical trials would be needed to establish the role of iodine in adjunct therapy of COVID-19. The role of iodine in the severe patients needed to be explored because of the higher mortality.

Positive results of the trial will equip the use of iodine to cope with COVID-19 in a relatively inexpensive and accessible way. Considering the pandemic nature of the disease, iodine can provide a global solution to mitigate the disease spread and severity especially in resource-limited countries like India.

## Trial status

Protocol Version Number is 2.3 and it is approved from IRB Shaikh Zayed Hospital with ID SZMC/IRB/Internal0056/2020 on July 14th, 2020. The recruitment is in progress. It was started on July 30, 2020, and the estimated trial completion date is February 15, 2022.

## Abbreviations

COVID-19: Coronavirus disease 2019
DM: Diabetes mellitus
DNA: Deoxyribose nucleic acid
FAIR: Findable, Accessible, Interoperable, Re-usable
HRCT: High-resolution computerized tomography
HTN: Hypertension
IRB: Institutional Review Board
RT-PCR: Real time polymerase chain reaction
RNA: Ribose nucleic acid
SARS-CoV: Severe acute respiratory syndrome coronavirus
SARS-CoV-2: Severe acute respiratory syndrome coronavirus 2
SZH: Shaikh Zayed Hospital
UVAS: University of Veterinary & Animal Sciences
KEMU: King Edward Medical University
SZPGMI: Shaikh Zayed Post-Graduate Medical Institute
PVP-1: Povidone-iodine 1
